# DNA methylation epigenotype and clinical features of *NRAS*‐mutation(+) colorectal cancer

**DOI:** 10.1002/cam4.1061

**Published:** 2017-04-04

**Authors:** Kiyoko Takane, Kiwamu Akagi, Masaki Fukuyo, Koichi Yagi, Tadatoshi Takayama, Atsushi Kaneda

**Affiliations:** ^1^ Department of Molecular OncologyGraduate School of MedicineChiba UniversityChibaJapan; ^2^ Department of Digestive Surgery and PathologyNihon University School of MedicineTokyoJapan; ^3^ Division of Molecular Diagnosis and Cancer PreventionSaitama Cancer CenterSaitamaJapan; ^4^ Department of Gastrointestinal SurgeryGraduate School of MedicineThe University of TokyoTokyoJapan

**Keywords:** *BRAF* mutation, colorectal cancer, DNA methylation, *KRAS* mutation, *NRAS* mutation

## Abstract

Sporadic colorectal cancer (CRC) is classified into several molecular subtypes. We previously established two groups of DNA methylation markers through genome‐wide DNA methylation analysis to classify CRC into distinct subgroups: high‐, intermediate‐, and low‐methylation epigenotypes (HME, IME, and LME, respectively). HME CRC, also called CpG island methylator phenotype (CIMP)‐high CRC, shows methylation of both Group 1 markers (CIMP markers) and Group 2 markers, while IME/CIMP‐low CRC shows methylation of Group 2, but not of Group 1 markers, and LME CRC shows no methylation of either Group 1 or Group 2 markers. While *BRAF‐* and *KRAS‐*mutation(+) CRC strongly correlated with HME and IME, respectively, clinicopathological features of *NRAS*‐mutation(+) CRC, including association with DNA methylation, remain unclear. To characterize *NRAS*‐mutation(+) CRC, the methylation levels of 19 methylation marker genes (6 Group 1 and 13 Group 2) were analyzed in 61 *NRAS*‐mutation(+) and 144 *NRAS*‐mutation(−) CRC cases by pyrosequencing, and their correlation with clinicopathological features was investigated. Different from *KRAS*‐mutation(+) CRC,*NRAS*‐mutation(+) CRC significantly correlated with LME. *NRAS*‐mutation(+) CRC showed significantly better prognosis than *KRAS*‐mutation(+) CRC (*P *=* *3 × 10^−4^). *NRAS*‐mutation(+) CRC preferentially occurred in elder patients (*P *=* *0.02) and at the distal colon (*P *=* *0.006), showed significantly less lymph vessel invasion (*P *=* *0.002), and correlated with LME (*P *=* *8 × 10^−5^). DNA methylation significantly accumulated at the proximal colon. *NRAS*‐mutation(+) CRC may constitute a different subgroup from *KRAS*‐mutation(+) CRC, showing significant correlation with LME, older age, distal colon, and relatively better prognosis.

## Introduction

Cancer arises through accumulation of genomic and epigenomic alternations 1, 2. Comprehensive genomic analyses of colorectal cancer (CRC) have been reported. Several important molecular aberrations are involved in CRC development, with disruption of critical signaling cascades such as RAS/RAF/ERK, WNT, TP53, and TGF‐*β* pathways 3, 4, 5. Epidermal growth factor receptor (EGFR) is activated in about 80% of CRC 6, leading to activation of the downstream RAS/RAF/ERK signaling and playing a significant role in tumor progression 6, 7. Cetuximab, a monoclonal antibody blocking the interaction between EGFR and its ligands, inhibits the downstream RAS signaling cascade and ERK activation in CRC therapy, and another EGFR‐neutralizing antibody, panitumumab, is available 8. Price et al. showed that these agents provided similar survival benefit, with more than 50% of participants having an overall survival longer than 10 months 9.

Downstream of EGFR, activating mutations of RAS and RAF also contribute to CRC development 10. *BRAF* mutation is observed in 5–10% of CRC and is mostly accompanied with frequent DNA hypermethylation and microsatellite instability (MSI) due to aberrant methylation of the *MLH1* promoter 11, 12. *KRAS* mutation is more frequently observed in 35–40% of CRC 13, 14, 15, 16, 17. *KRAS*‐mutation(+) CRC reportedly shows worse prognosis, even under 5‐FU based chemotherapy 18, 19, 20. Targeting the EGFR using EGFR‐neutralizing antibodies is ineffective for treatment of CRC with mutation of these oncogenes because the RAS/RAF/ERK signaling cascade is downstream of EGFR 21, 22. *NRAS* mutation occurs infrequently at 2.6–4.2% 23, 24, 25, 26, 27, 28, 29, 30, 31, 32. *NRAS*‐mutation(+) CRC prognosis remains controversial, since only few studies on its prognosis, analyzing 4–73 cases, have been reported 23, 24, 25, 26, 27, 28, 29, 30, 31, 32, 33. While Gavin et al. reported no significant difference in prognosis between 73 *NRAS*‐mutation(+) and 750 *KRAS*‐mutation(+) cases 26, other groups studied 4–35 *NRAS*‐mutation(+) cases and did neither reveal significant difference in prognosis nor conduct prognosis analysis 23, 24, 25, 27, 28, 29, 30, 31, 32.

We and others previously stratified CRC using comprehensive and quantitative DNA methylation data 18, 34. In 1999, Toyota et al. reported CRC subtypes with frequent CpG island hypermethylation, so‐called CpG island methylator phenotype (CIMP) 11. In 2010, Yagi et al. established two groups of methylation marker genes, Group 1 and Group 2 markers, to classify CRC into three distinct epigenotypes: high‐, intermediate‐, and low‐methylation epigenotypes (HME, IME, and LME, respectively) 18. While Group 1 markers are mostly equivalent to classical CIMP markers 11, 12, 35, 36, HME/CIMP‐high CRC shows methylation of both Group 1 and Group 2 markers, and strongly correlate with *BRAF*‐mutation(+). IME/CIMP‐low CRC shows methylation of Group 2, but not Group 1 markers, and strongly correlates with *KRAS*‐mutation(+). LME CRC shows no methylation of Group 1 and Group 2 markers, and do not correlate with mutations in these oncogenes 18, 37.

However, molecular features of *NRAS*‐mutation(+) CRC, including DNA methylation epigenotype and its clinicopathological features, are largely unknown. We therefore investigated DNA methylation levels in 61 *NRAS*‐mutation(+) CRC and 144 *NRAS*‐mutation(−) CRC samples using 6 Group 1 and 13 Group 2 methylation markers by pyrosequencing to characterize epigenetic features of *NRAS*‐mutation(+) CRC and analyzed its clinicopathological features.

## Materials and Methods

### Clinical samples

A total of 2045 CRC samples were obtained from CRC patients who underwent surgery at Saitama Cancer Center with written informed consent, and kept frozen until use. Among the 2045 CRC samples, 61 cases were positive for *NRAS* mutation, based on mutation analysis described below. In addition to these 61 *NRAS*‐mutation(+) CRC samples, 144 *NRAS*‐mutation(−) CRC samples, including 70 cases whose epigenotypes were already estimated in our previous study (10 HME, 30 IME, and 30 LME) 14, underwent subsequent analyses. CRC specimens were microscopically examined for determination of cancer cell contents by two independent pathologists. Samples that contained at least 40% of cancer cells were used for the subsequent analyses, and the specimens were dissected to enrich cancer cells when necessary. DNA was extracted using QIAamp DNA Micro Kit (Qiagen, Hilden, Germany). Their clinicopathological features, for example, age, gender, tumor location, mucinous component, and tumor stage based on American Joint Committee on Cancer (AJCC), lymph node metastasis, lymph vessel invasion, venous invasion, and microsatellite instability, are summarized in Table 1. This study was certified by the Ethics Committee of Chiba University and Saitama Cancer Center.

**Table 1:** Comparison of clinicopathological features of CRC excluding stage I cases

Clinical features	All cases	*BRAF*	*KRAS*	*NRAS*	No‐mut	*P‐*value (*K* vs. *N* vs. No)	*P*‐value (*K* vs. *N*)
Number of samples	186	10	59	45	72		
Gender							
Male	110	9	33	20	48	0.2	0.7
Female	70	1	26	19	24		
Unknown	6	0	0	6	0		
Age (y.o.)							
Mean ± SD	63.8 ± 9.4	72.0 ± 8.6	61.7 ± 9.4	66.0 ± 9.2	62.0 ± 9.1	0.04^a^	0.02^a^
Tumor location							
Proximal	55	10	23	5	17	0.01^a^	0.006^a^
Distal	124	0	36	33	55		
Unknown	7	0	0	7	0		
Mucinous component							
(+)	27	6	14	2	5	0.005^a^	0.02^a^
(−)	152	4	45	36	67		
Unknown	7	0	0	7	0		
AJCC stage							
I	54	5	17	16	16	0.3	0.5
III	61	3	21	11	26		
IV	65	2	21	12	30		
Unknown	6	0	0	6	0		
Lymph node metastasis							
(+)	104	5	39	17	43	0.2	0.09
(−)	72	5	20	19	28		
Unknown	10	0	0	9	1		
Lymph vessel invasion							
(+)	130	10	48	19	53	0.003^a^	0.002^a^
(−)	49	0	11	19	19		
Unknown	7	0	0	7	0		
Venous invasion							
(+)	149	8	48	31	62	0.7	1.0
(−)	30	2	11	7	10		
Unknown	7	0	0	7	0		
Microsatellite instability							
MSI‐H	14	8	3	0	3	0.4	0.3
MSS	164	2	56	37	69		
Unknown	8	0	0	8	0		
Methylation epigenotype							
HME	10	6	2	0	2	2 × 10^−4^ a	8 × 10^−5^ a
IME	84	4	40	13	27		
LME	92	0	17	32	43		

*No‐mut*, no mutation; *K* vs. *N* vs. *No*,* KRAS* versus *NRAS* versus no mutation; *K* vs. *N*,* KRAS* versus *NRAS*; MSI‐H, microsatellite instability high; MSS, microsatellite stable; HME, high‐methylation epigenotype; IME, intermediate‐methylation epigenotype; LME, low‐methylation epigenotype.

a *P *<* *0.05

### Mutation analysis

*KRAS* mutation (codons 12, 13, and 19) and *NRAS* mutation (codon 12, 13, 59, and 61) were analyzed as described previously 18, 38. *BRAF* mutation (V600E) at exon 15 was determined by direct sequencing using pyrosequencing as previously reported 39. The cut‐off value for positive result of mutation was set at 20% on the sequencer, considering the tumor cell content (≥40%).

### Bisulfite treatment

Bisulfite conversion of 500 ng of genomic DNA from each tissue sample was performed using Zymo EZ DNA Methylation Kit (Zymo Research, Irvine, CA), and the DNA was eluted in 80 *μ*L of 10 mEq Tris buffer. By bisulfite treatment, unmethylated cytosine is converted to uracil, that is, recognized as thymine (T) after PCR, but methylated cytosine is not converted, that is, cytosine (C) after PCR. Unmethylated DNA and methylated DNA are therefore distinguishable by detecting the difference of T and C in the sequence after bisulfite treatment.

Methylation control samples (0%, 25%, 50%, 75%, and 100%) were prepared as described previously 18. Briefly, human peripheral lymphocyte DNA was amplified using GenomiPhi v2 DNA amplification kit (GE Healthcare Life‐Science, Buckinghamshire, UK). The amplified DNA was not methylated in any CpG sites, and was used as unmethylated (0%) control. The amplified DNA was methylated by *Sss*I methylase and used as fully methylated (100%) control. Other methylation control samples (25%, 50%, and 75%) were prepared by mixing 0% and 100% samples at a ratio of 3:1, 1:1, and 1:3. These control samples were also treated with bisulfite in the same manner.

### Methylation analysis

Quantitative methylation analysis was performed by pyrosequencing as previously reported 37, 40. Briefly, the biotinylated PCR product was bound to Streptavidin Sepharose High Performance (Amersham Biosciences, Uppsala, Sweden), washed, and denatured using a 0.2 mol/L NaOH solution. After addition of 0.3 *μ*mol/L sequencing primer to the single‐stranded PCR product, pyrosequencing was carried out according to the manufacturer's instructions. By using methylation control samples (0%, 25%, 50%, 75%, and 100%), it was confirmed in each pyrosequencing assay that methylation analysis for the 19 markers was done highly quantitatively. Primer sequences of the methylation markers are shown in Table S1.

### Statistical analysis

The clinicopathological features were compared between *BRAF*‐mutation(+), *KRAS*‐mutation(+), *NRAS*‐mutation(+), and oncogene‐mutation(−) CRC groups. *P*‐value was calculated by the Student's *t*‐test for age and methylation level, by chi‐square test for AJCC stage, and by Fisher's exact test for gender, tumor location, mucinous component, lymph node metastasis, lymph vessel invasion, venous invasion, and microsatellite instability, using R software (https://www.r-project.org/). Unsupervised two‐way hierarchical clustering was performed based on the City‐block distance, the complete linkage‐clustering algorithm using Cluster 3.0 software. The heatmap was drawn using Java Tree View software. In survival analysis, Kaplan–Meier survival curve was drawn by GeneSpring 7.3.1 software, and *P*‐value was calculated by log‐rank test and Fisher's exact test. The end of the follow‐up period was 60 months from the primary surgery, and the death because of CRC was the primary endpoint; deaths by other causes were censored. Survival analysis by Cox proportional hazard model was also performed using R software. Correlation of the methylation level of each marker with tumor location and age was evaluated by linear single regression model using R software.

## Result

### Oncogene mutation analysis

Among 2045 CRC cases, 61 cases were positive for *NRAS* mutation. These and the additional 144 *NRAS*‐mutation(−) CRC cases were analyzed for *BRAF* and *KRAS* mutations. The 205 CRC cases included 13 cases with *BRAF* mutation, 59 with *KRAS* mutation only, 56 with *NRAS‐*mutation only, 5 with both *KRAS* and *NRAS* mutations, and 72 with no mutation of these oncogenes (Table S2).

### Quantitative DNA methylation analysis

**Figure 1:**
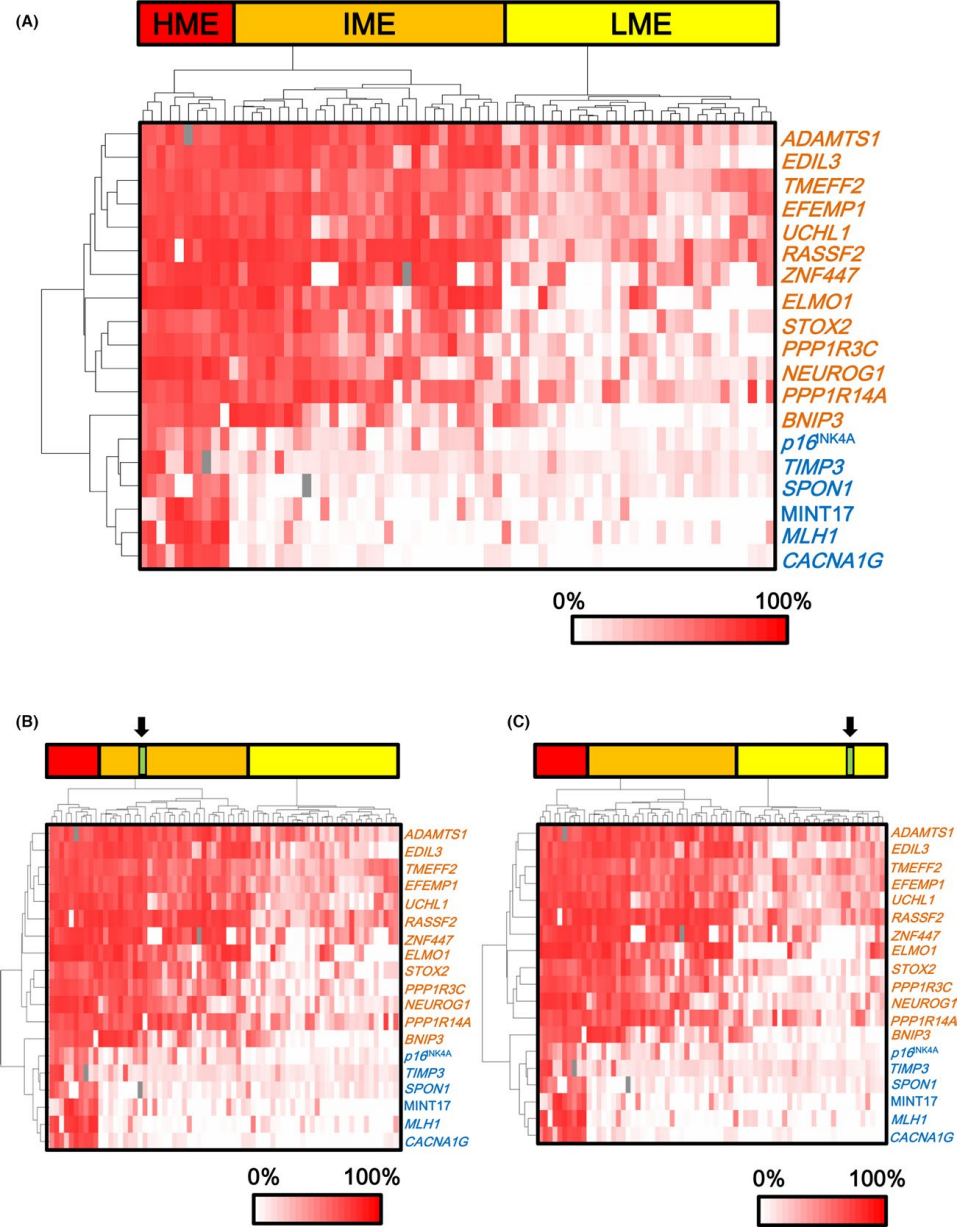
Hierarchical clustering of sporadic CRC samples. (A) Hierarchical clustering of 70 CRC samples. To evaluate the methylation epigenotype of CRC samples by comparing the previously established methylation epigenotypes of sporadic CRC, we used the cluster of 70 sporadic CRC samples, including 10 high‐, 30 intermediate‐, and 30 low‐methylation epigenotypes 18. *Blue*: Group 1 markers, including *p16INK4A*,*TIMP3*,*SPON1*, MINT17, *MLH1*, and *CACNA1G. Orange*: Group 2 markers, including *ADAMTS1*,*TMEFF2*,*STOX2*,*COLA4A2*,*EDIL3*,*UCHL1*,*RASSF2*,*ELMO1*,*PPP1R3C*,*PPP1R14A*,*BNIP3*,*ZNF447*, and *NEUROG1*. (B) One CRC sample belonging to IME. *Arrow*: One CRC sample. (C) One CRC sample belonging to LME. *Arrow*: One CRC sample.

Methylation levels of six Group 1 and 13 Group 2 markers were quantitatively analyzed by pyrosequencing. Hierarchical clustering analysis was conducted using methylation data of these 19 markers to evaluate methylation epigenotype. First, we performed hierarchical clustering analysis using 70 CRC samples whose epigenotypes was previously evaluated (10 HME, 30 IME, and 30 LME) 18 (Fig. 1A). The 70 CRC samples were properly classified into three distinct clusters using the 19 markers. Second, we performed hierarchical clustering analysis of each CRC sample with the 70 CRC samples (Fig. 1B and C). Three CRC samples were clustered with 10 HME samples, 61 were clustered with 30 IME samples (Fig. 1B), and 71 were clustered with 30 LME samples (Fig. 1C).

**Figure 2:**
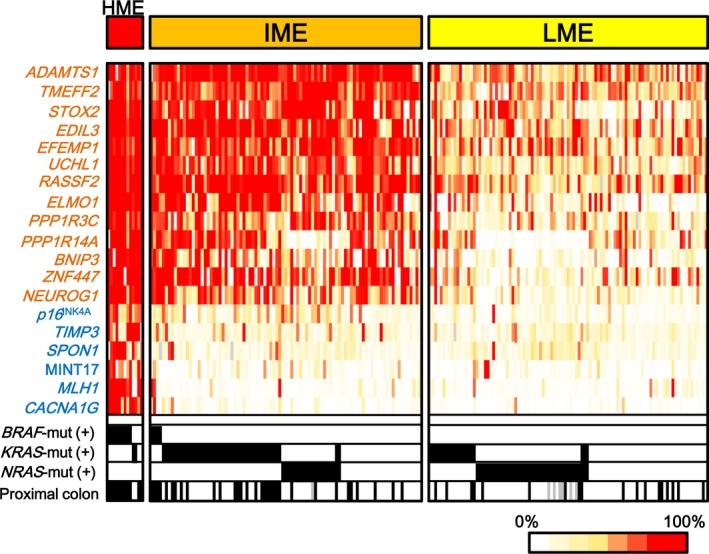
*NRAS*‐mutation(+) CRC showed two major subtypes. After evaluating methylation epigenotype of CRC samples, all CRC samples were divided into three groups. While none of *NRAS*‐mutation(+) cases was equivalent to HME, 20 *NRAS*‐mutation(+) CRC were equivalent to IME CRC and 41 cases were equivalent to LME CRC (*P *=* *0.008). *NRAS‐mut(+)*,*KRAS‐mut(+)*, or *BRAF‐mut(+)*: samples positive for *NRAS*‐mutation, *KRAS*‐mutation, or *BRAF*‐mutation are shown in *black*. *Blue*: Group 1 markers. *Orange*: Group 2 markers.

The 61 *NRAS*‐mutation(+) CRC were classified into two major subtypes (Fig. 2). While none of the *NRAS*‐mutation(+) CRC was evaluated as HME, 20 *NRAS*‐mutation(+) CRC were evaluated as IME and 41 were LME. In contrast, *KRAS*‐mutation(+) cases were mostly IME (40 of 59), and *BRAF*‐mutation(+) cases were mostly HME (10 of 13) (*P *=* *1 × 10^−4^, chi‐square test). These data indicated that *NRAS*‐mutation(+) CRC preferentially showed LME, while *KRAS*‐mutation(+) CRC strongly correlated with IME (Table S2).

### Comparison of the methylation level of each marker

**Figure 3:**
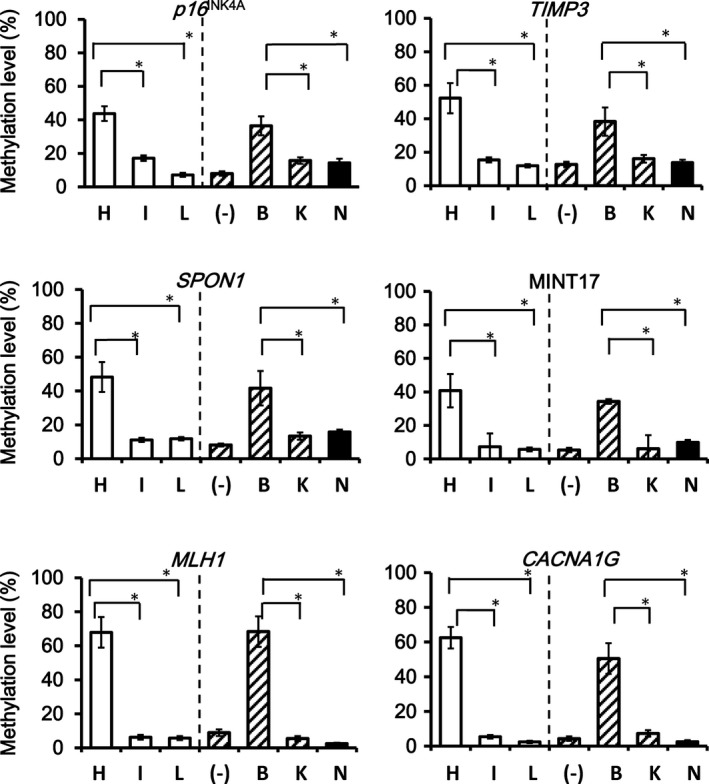
Comparison of the methylation level of Group 1 markers. All Group 1 markers showed that HME CRCs presented higher methylation levels than LME and IME CRC. Similarly, all the Group 1 markers showed significantly higher methylation levels in *BRAF*‐mutation(+) CRC than in *KRAS*‐mutation(+) and *NRAS*‐mutation(+) CRC (*P <* *0.05).

**Figure 4:**
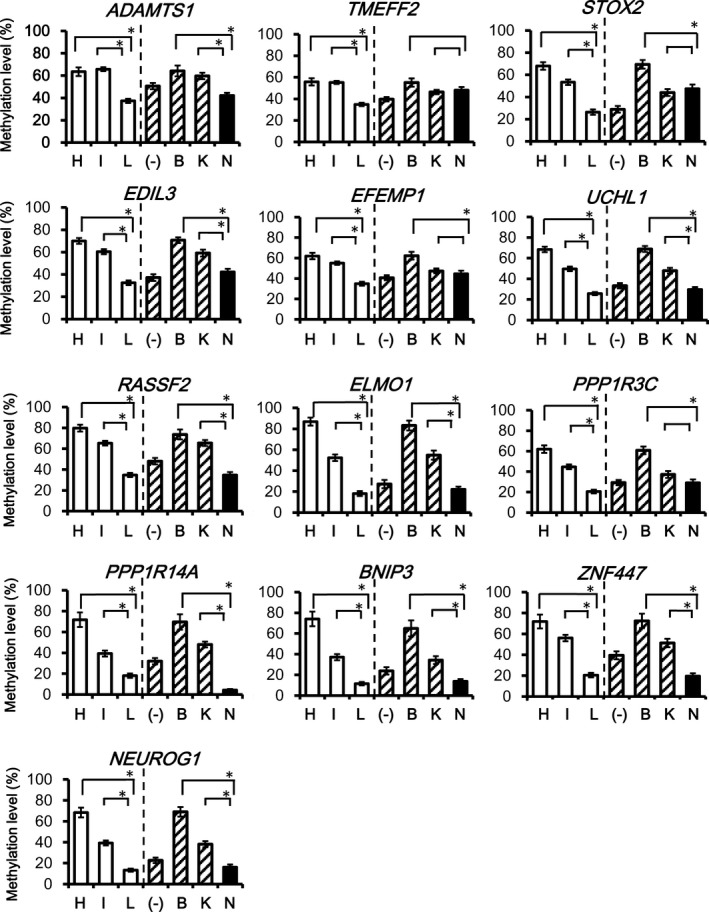
Comparison of the methylation level of Group 2 markers. While 12 of 13 Group 2 markers showed significantly lower methylation levels in *NRAS*‐mutation(+) CRC than in *BRAF*‐mutation(+) CRC, all Group 2 markers showed significantly lower methylation level in LME CRC than in HME CRC. Additionally, while 9 of 13 Group 2 markers presented low methylation in *NRAS*‐mutation(+) CRC than in *KRAS*‐mutation(+) CRC, all Group 2 markers showed significantly lower methylation levels in LME CRC than in IME CRC (*P <* *0.05).

To confirm that *NRAS*‐mutation(+) CRC preferentially showed LME, not IME, methylation levels of each marker were compared among the three epigenotypes, and among mutation types (Figs. 3 and 4).

All six Group 1 markers showed significantly higher methylation levels in HME CRC than that in LME and IME CRC. Similarly, all Group 1 markers showed significantly higher methylation levels in *BRAF*‐mutation(+) CRC than *KRAS*‐mutation(+) and *NRAS*‐mutation(+) CRC (*P *<* *0.05) (Fig. 3).

As for Group 2 markers, methylation levels in LME CRC were significantly lower than that in HME and IME in all genes (*P *<* *0.05) (Fig. 4). Similarly, in all genes, except *TMEFF2*, methylation levels in *NRAS*‐mutation(+) CRC were significantly lower than that in *BRAF*‐mutation(+) CRC. For nine of 13 Group 2 markers, methylation levels in *NRAS*‐mutation(+) CRC were significantly lower than that in *KRAS*‐mutation(+) CRC.

Consistent with the clustering analysis, significantly lower levels of Group 1 and Group 2 markers indicated that *NRAS*‐mutation(+) CRC correlated with LME.

### Comparison of clinocopathological features and mutation types

The clinicopathological data of the 205 analyzed CRC cases are summarized in Table S2. *NRAS*‐mutation(+) CRC significantly correlated with older age, distal colon, more mucinous component of the tumor, earlier AJCC stage, less lymph node metastasis, and less lymph vessel invasion (*P *=* *0.01, 0.002, 0.02, 0.001, 0.003, and 8 × 10^−6^, respectively), compared with *KRAS*‐mutation(+) CRC. Since *NRAS*‐mutation(+) CRC included cases with earlier AJCC stage, we excluded stage I CRC cases and performed similar analyses using 186 cases with stage II–IV (Table 1). While *NRAS*‐mutation(+) CRC and *KRAS*‐mutation(+) CRC did not show significant difference in term of AJCC stage in this analysis (*P *=* *0.5), *NRAS*‐mutation(+) CRC still significantly correlated with older age, distal colon, more mucinous component of the tumor, and less lymph vessel invasion (*P *=* *0.02, 0.006, 0.02, and 0.002, respectively).

### Survival analysis of CRC patients

**Figure 5:**
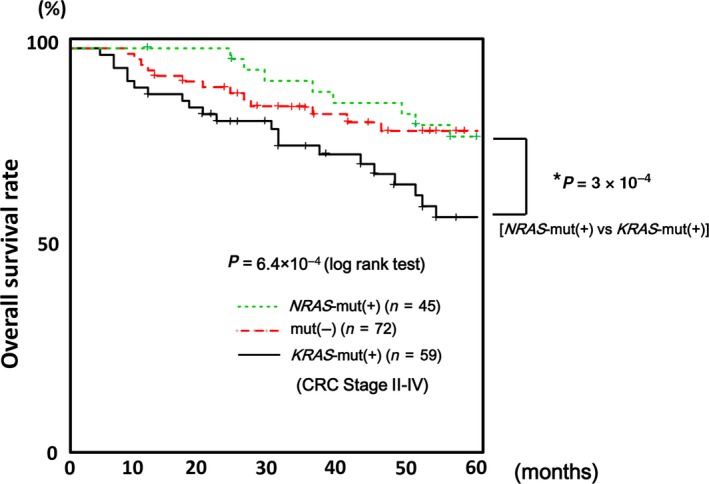
Kaplan–Meier analysis of survival in CRC, excluding stage I. Kaplan–Meier survival analysis showed significant differences (*P *=* *6 × 10^−4^) among *NRAS*‐mutation(+), *KRAS*‐mutation(+), and oncogene‐mutation(−) groups. Compared with *NRAS*‐mutation(+) and *KRAS*‐mutation(+) groups, *NRAS*‐mutation(+) CRC did not show worse prognosis in overall survival (*P *=* *3 × 10^−4^, log‐rank test).

We then conducted an overall survival analysis using these stage II–IV CRC cases. Kaplan–Meier survival analysis showed significant differences among *NRAS*‐mutation(+), *KRAS*‐mutation(+), and oncogene‐mutation(−) groups (*P *=* *6 × 10^−4^, log‐rank test) (Fig. 5). Comparison of *NRAS*‐mutation(+) and *KRAS*‐mutation(+) groups, indicated a significantly better prognosis for *NRAS*‐mutation(+) CRC (*P *=* *3 × 10^−4^, log‐rank test). When overall survival rates at 60 months were compared among *NRAS*‐mutation(+), *KRAS*‐mutation(+), and oncogene‐mutation(−) groups, *NRAS*‐mutation(+) and oncogene‐mutation(−) CRC also showed better survival rates than *KRAS*‐mutation(+) CRC (*P *=* *0.02, chi‐square test) (Table 2). When analyzing the correlation of overall survival rates with other clinicopathological features (Fisher's exact test, *t*‐test, and chi‐square test), higher AJCC stage (*P *=* *2 × 10^−5^), lymph node metastasis (*P *=* *0.004), and venous invasion (*P *=* *0.04) were also correlated with worse survival rate at 60 months. To identify independent prognosis factors, multivariate overall survival analysis was conducted by Cox proportional hazard model. *RAS* status and AJCC stage were statistically significant (*P *=* *0.007 and 0.02, respectively), while other factors were not (Table 3).

**Table 2:** Prognosis at 60 months and comparison with clinicopathological features

Clinical features	Death (*n* = 42)	Alive (*n* = 65)	*P*‐value
RAS			
* NRAS*	8	26	0.02^a^
* KRAS*	21	17	
No‐mut	13	22	
Gender			
Male	26	35	0.4
Female	16	30	
Age (y.o.)	62.1 ± 9.6	64.0 ± 9.0	0.3
Tumor location			
Proximal	11	15	0.8
Distal	31	50	
AJCC stage			
II	5	21	2 × 10^−5^ a
III	6	26	
IV	31	18	
Mucinous component			
(+)	4	9	0.6
(−)	38	56	
Lymph node metastasis			
(+)	34	34	0.004^a^
(−)	8	31	
Lymph vessel invasion			
(+)	33	53	0.8
(−)	9	12	
Venous invasion			
(+)	40	53	0.04^a^
(−)	2	12	
Microsatellite instability			
MSI‐H	0	2	0.5
MSS	42	63	
Methylation epigenotype			
HME	2	2	0.3
IME	23	27	
LME	17	36	

*No‐mut*, no mutation; MSI‐H, microsatellite instability high; MSS, microsatellite stable; HME, high‐methylation epigenotype; IME, intermediate‐methylation epigenotype; LME, low‐methylation epigenotype.

a *P *<* *0.05

**Table 3:** Multivariate overall survival analysis by Cox proportional hazard model

Clinicopathological features	*P*‐value
RAS	0.007^a^
Gender	0.4
Age (y.o.)	0.6
Tumor location	0.4
AJCC stage	0.02^a^
Mucinous component	0.5
Lymph node metastasis	0.5
Lymph vessel invasion	0.3
Venous invasion	0.2
Microsatellite instability	0.9
Methylation epigenotype	0.9

a *P *<* *0.05.

### Comparison using linear single regression

**Figure 6:**
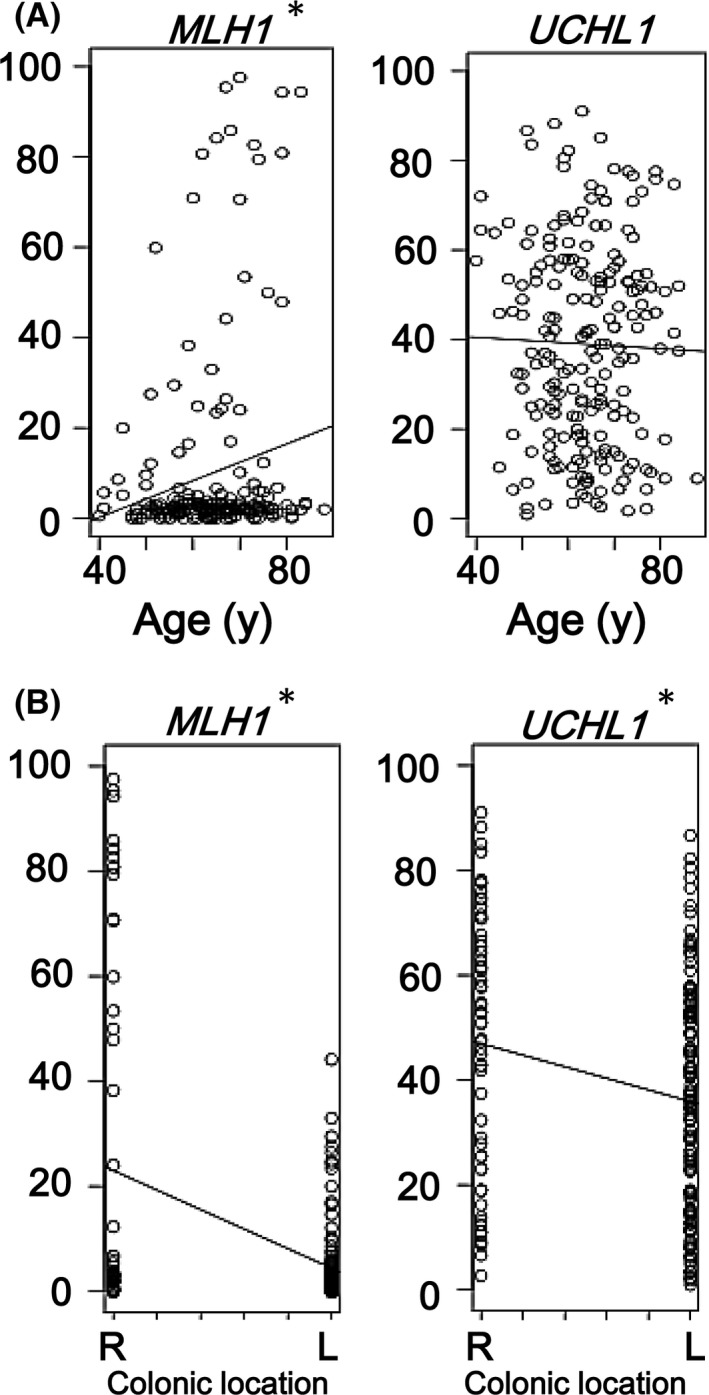
Comparison using linear single regression model. (A) Association of methylation accumulation with age. CRC samples of elder patients showed significantly higher methylation levels in Group 1 markers, for example., *MLH1*, than in Group 2 markers, for example., *UCHL1* (see Fig. S1). (B) Association of methylation accumulation with location. Group 2 markers, for example., *UCHL1*, and Group 1 marker, for example., *MLH1*, showed significant correlation between higher methylation levels and proximal location (Fig. S2). Since 6 Group 1 markers and 13 Group 2 markers were evaluated for each factor, *P* < 0.008 (i.e., 0.05/6) and *P* < 0.004 (i.e., 0.05/13) were considered significant in the analysis of Group 1 and Group 2 markers, respectively, instead of *P *<* *0.05 (*).

Correlation between DNA methylation levels and clinicopathological factors other than oncogene mutation status, for example, tumor location and age, was analyzed using linear single regression model (Fig. 6). A significant correlation was observed between higher methylation level and older age for three of six Group 1 markers, but not for any Group 2 markers (Figs. 6A and S1). A significant correlation was observed between higher methylation level and proximal colon for five of six Group 1 markers and 10 of 13 Group 2 markers (Figs. 5B and S2).

## Discussion

The RAS pathway plays an important role in the development of various cancers 41, 42, 43. As one of the RAS family members, *NRAS* contains effector binding domains identical to those in *KRAS*. Thus, *NRAS* activating mutations yield effects similar to those observed after *KRAS* activation 44. Patients with *KRAS* and *NRAS* mutations are resistance to anti‐EGFR monoclonal antibody therapy. The PRIME trial showed a detrimental effect of adding panitumumab to first‐line FOLFOX in patients with RAS mutations 45. De Roock et al. evaluated the role of *NRAS* mutations in a large dataset of chemorefractory patients with CRC treated with cetuximab and chemotherapy in 11 centers in seven European countries. Only one RECIST response was reported among 13 patients with *NRAS* mutations in this retrospective series 46. Peeters et al. reported that none of the 11 patients with *NRAS*‐mutation(+) responded to panitumumab in a randomized phase III study compared to best supportive care 47. Considering the resistance to EGFR targeted therapy, the molecular basis and clinicopathological features of patients with *NRAS*‐mutation(+) CRC should be analyzed and clarified as previously performed for *KRAS*‐mutation(+) CRC.

However, *NRAS* mutations are observed infrequently in 2.6–4.2% CRC in sporadic CRC, while *KRAS* mutations are frequently observed in 35–40% CRC. The Cancer Genome Atlas conducted a comprehensive analysis of human CRC, but the study included only 20 *NRAS*‐mutation(+) CRC cases and revealed no correlation with CIMP or methylation of any genes 4. Here, we analyzed 61 *NRAS*‐mutation(+) and 144 *NRAS*‐mutation(−) CRC, and identified that, while *BRAF* mutation and *KRAS* mutation significantly correlated with HME and IME, *NRAS* mutation significantly correlated with LME, a different DNA methylation epigenotype.

In addition to epigenetic features, *NRAS*‐mutation(+) CRC also showed different clinicopathological features compared to *KRAS*‐mutation(+) CRC. *NRAS*‐mutation(+) CRC significantly correlated with older age, distal colon, more mucinous component of the tumor, and lower lymph vessel invasion when compared with *KRAS*‐mutation(+) CRC. The comparison of 73 *NRAS*‐mutation(+) and 750 *KRAS*‐mutation(+) cases by Gavin et al. 26 or that of 43 *NRAS*‐mutation(+) and 504 *KRAS*‐mutation(+) cases by Zhang et al. 30, revealed no significant difference in these parameters. Ogura et al. analyzed 35 *NRAS*‐mutation(+) CRC and found that *NRAS*‐mutation(+) CRC significantly correlated with older age comparing with *KRAS*‐mutation(+) CRC 23. While Schirripa et al. 33 reported lower prevalence of mucinous histology in 47 *NRAS*‐mutation(+) CRC compared with 393 *KRAS*‐mutation(+) CRC, this is the first report revealing the significant difference of the above clinicopathological features between *NRAS*‐mutation(+) CRC and *KRAS*‐mutation(+) CRC.

Although *KRAS* and *BRAF* mutations are both aberrations of the RAS/RAF/ERK pathway downstream of EGFR, they correlated with distinct DNA methylation epigenotypes and clinicopathological features, suggesting different molecular pathways of tumorigenesis 18, 48. In fact, *BRAF*‐mutation(+) CRC is mostly HME/CIMP‐high, MSI‐high CRC, whose precursor lesions are sessile serrated adenoma/polyps preferentially occurring at the proximal colon 49. Similarly, the different epigenetic and clinicopathological features of *NRAS*‐mutation(+) CRC may suggest a different tumorigenic pathway through different types of early lesions. While *KRAS* gene is located on the short (p) arm of chromosome 12 and *NRAS* is located on the chromosome 1 at position 13.1, the protein localization is different 50, 51. Oncogenic NRAS colocalizes with markers of the endoplasmic reticulum and Golgi 52. While the oncogenic NRAS is restricted to the endoplasmic reticulum with a transmembrane tether and retained transforming activity in a focus‐forming assay, NRAS restricted to the Golgi is unable to promote transformation 53. On the other hand, KRAS is targeted to the plasma membrane by an uncharacterized pathway and returns to endomembrane compartments following phosphorylation of the hypervariable region 54. Furthermore, *NRAS* and *KRAS* mutations may have different effects on cell biology. Haigis et al. found that activated KRAS affects cell proliferation and differentiation, whereas activated NRAS suppresses apoptosis 55. *NRAS* mutation does not seem to affect the early phases of tumor progression and the adenoma–carcinoma sequence, but it might inhibit epithelial cells’ stress‐induced apoptosis 55. A different effect of these mutations on downstream signaling cascade effectors has also been hypothesized 56. Wang et al. found that mutant NRAS strongly promotes tumorigenesis in the context of inflammation 56. In addition, mutations in different RAS genes are preferentially associated with distinct tumor types in human cancers. *KRAS* mutations are extremely common in cancer of the pancreas, colon, and lung, while *NRAS* mutations predominate in melanoma and hematopoietic cancers 57. To clarify whether *NRAS*‐mutation(+) CRC constitutes a unique CRC subtype occurring through distinct tumorigenic pathway, further analyses should be performed, including comprehensive analyses of genomic and epigenomic aberrations in precancerous *NRAS*‐mutation(+) colorectal lesions, for example, aberrant crypt foci and adenoma as well as *NRAS*‐mutation(+) CRC.

The prognosis and degree of malignancy of *NRAS*‐mutation(+) CRC is controversial. *NRAS*‐mutation(+) CRC shows a high degree of malignancy compared with oncogene‐mutation(−) CRC 33, and there is no significant difference in prognosis of *NRAS*‐mutation(+) and *KRAS*‐mutation(+) CRC 23, 26, 33. However, in this study, Kaplan–Meier survival analysis showed that, while *KRAS*‐mutation(+) CRC showed significantly worse prognosis, *NRAS*‐mutation(+) CRC and oncogene‐mutation(−) CRC showed relatively better prognosis. Survival analysis by Cox proportional hazard model showed that RAS status and AJCC stage were independent prognosis determining factors (*P *=* *0.007 and 0.02, respectively).

To evaluate the possible association of methylation accumulation with tumor location and age, methylation levels were analyzed by a linear single regression model using all sporadic CRC samples (Figs. 6, S1, and S2). DNA methylation accumulated significantly more in the proximal colon than in the distal colon for most of Group 1 and Group 2 markers. Since sessile serrated adenoma/polyps are known as the precursor lesions of HME/CIMP‐high CRC and preferentially observed at the proximal colon 28, 49, we performed similar analyses using IME and LME CRC cases only (Fig. S3). A significant correlation was still observed between higher methylation level and proximal colon for 2 of 6 Group 1 markers and 7 of 13 Group 2 markers. Thus, DNA methylation significantly accumulated in the proximal colon, regardless of HME/CIMP‐high samples. Aging is known as an important factor causing DNA methylation accumulation 58. While three of six Group 1 markers (classical CIMP markers) showed a significant correlation between higher methylation level and older age, no correlation was observed in any Group 2 markers. Further analyses are necessary to identify factors that induce DNA methylation in Group 2 markers.

In summary, *NRAS*‐mutation(+) CRC showed distinct epigenetic and clinicopathological features. *NRAS*‐mutation(+) CRC significantly correlated with LME, while *KRAS*‐mutation(+) CRC correlated with IME. *NRAS*‐mutation(+) CRC significantly correlated with less lymph vessel invasion, occurred preferentially in elder patients and at the distal colon, and showed relatively better prognosis, compared with *KRAS*‐mutation(+) CRC.

## Conflict of Interest

All authors have no potential conflict of interest to disclose.

## Supporting information


**Table S1.** Methylation marker genes and printer sequences for pyrosequences.
**Table S2.** Comparison of clinicopathological features of all CRC cases.
**Figure S1**. Comparison between methylation levels and age using linear single regression model.
**Figure S2.** Comparison between methylation levels and tumor location using a linear single regression model.
**Figure S3.** Comparison between methylation levels and tumor location using a linear single regression model, excluding HME CRCs.
