# Supplementary material for: DNA methylation epigenotype and clinical features of NRAS‐mutation(+) colorectal cancer
Source: Cancer Med. 2017 Apr 4;6(5):1023–35. doi: 10.1002/cam4.1061 (PMC5430106; doi:10.1002/cam4.1061)
Supplement: Supplementary file 1 — Table S1. Methylation marker genes and printer sequences for pyrosequences. Table S2. Comparison of clinicopathological features of all CRC cases. Figure S1. Comparison between methylation levels and age using linear single regression model. Figure S2. Comparison between methylation levels and tumor location using a linear single regression model. Figure S3. Comparison between methylation levels and tumor location using a linear single regression model, excluding HME CRCs. [file CAM4-6-1023-s001.pdf]

Supplementary information

DNA methylation epigenotype and clinical features of NRAS-mutation(+) colorectal cancer

Kiyoko Takane MD, PhD, Kiwamu Akagi MD, PhD, Masaki Fukuyo PhD, Koichi Yagi MD, PhD, Tadatoshi Takayama MD, PhD, Atsushi Kaneda MD, PhD

Supplementary Tables:

Supplementary Table S1.

Supplementary Table S2.

Supplementary Figures:

Supplementary Figure S1.

Supplementary Figure S2.

Supplementary Figure S3.

Supplementary Table S1. Methylation marker genes and primer sequences for pyrosequencing

Gene		PCR Primers (Forward/Reverse)	Sequencing primers
**Group-1**			
SPON1	**F**	AGGAGGTTGTTGTTTTAATTTTTAGTTATA	AGTTATATTTAGGATTTTTTGGAG
	**R***	AATTATTTCTCAAACTTTCCCTCCTCTA	
TIMP3	**F**	AGAGATATTTAGTGGTTTAGGTG	GGTGGGAGTGGGGTTA
	**R***	AAACCCTCCTACCCCTTCT	
CACNA1G	**F**	TGTTTTGGTTTAAGTAGAAGAAAAT	TGGTTTAAGTAGAAGAAAATTAT
	**R***	ACCCAAATCAACAAAAAAAAACCC	
MLH1	**F**	ATGTGGATGAAGTTTAAAAGAAGTAAGAT	ATGGAAGTAGAAGAGGTTTTAGTTT
	**R***	AAAACTCCACCACCAAATA	
MINT17	**F***	AAGTGGGAGAAGAGGAAGAGAAAAAATA	TATCCCTCCCCATCT
	**R**	CTATCCTCCCCAAACTTCT	
p16INKA	**F***	GGTGGGTAGAGGGTTTGTA	GGGAGTAGGGGATGG
	**R**	CCAATTCCCCTACAAACTT	
**Group-2**			
ADAMTS1	**F**	GTTTTTTGGGGTTTTAATGTAG	TTAATGTAGAGAGTTGTGTT
	**R***	TTCCCCATATCCCTACCCAACTTAC	
BNIP3	**F**	AGAAGTAGATTTATTTTTAGGTGGAATT	ATTTTTAGGTGGAATTTTAGT
	**R***	AAAAAACCCCCATTCTCCAACT	
EDIL3	**F**	GGGATTTTTAGTTTATTTTTTATTTAGTTG	TTAGTTTATTTTTTATTTAGTTGTT
	**R***	ACTCAACCTCCATATCCCCCAATT	
EFEMP1	**F***	TTGGGAAGTTGAGTAGTTTTAGGG	AAATCCCCTTTCTTAACA
	**R**	ACCCCACAAAAAAATAAAATCCCCTTTCTT	
ELMO1	**F**	GGAGGAAGGAAGAGGAAGTGA	GAGGAAGTGAGAGTAG
	**R***	CCAACACACCCACTTACACTCTAAAT	
PPP1R3C	**F**	GTGTTTGGGAGTAGATAAG	TTTTTAGGAGTAGGGTTTTTAGTTT
	**R***	CACAACTCCAAACCTTACC	
PPP1R14A	**F**	GGGGGTTTGGGATAGATAT	GGGATTAGGTTTGTTTG
	**R***	CTCCCCCACCAACAACCC	
RASSF2	**F**	AGGGTTGTTAGGTTTTTTTTAGT	GTTAGGTTTTTTTTAGTTTTGTA
	**R***	CCCATCCCCCAAATCTCTAAAACTT	
STOX2	**F**	AGGTTGGGGTAGTTGTTAAG	GGGTAGTTGTTAAGGTTT
	**R***	TCCCATCAAACTTCTCATTTTCA	
TMEFF2	**F**	ATTTAGGGATTGGGTTTAGT	AGGGTAGTTAGTTGAGAAGT
	**R***	CCCTCCTTATAACAACAACT	
UCHL1	**F***	GGTAGGGTTTTTAAATTTTGTAGTTTTATT	CCACCAAATTATCTCACC
	**R**	CCACAACCACCAAATTATCT	
ZNF447	**F**	GGGGTAGTTGAGTAGTAGGTGG	GGTAGGTTTAGGGGATGTAG
	**R***	CCTCACCCTCTACCCTATTAAAATC	
NEUROG1	**F**	AGTTTGGGGTTGTTATTTTGTGTTA	GTTGTTATTTTGTGTTAGTTG
	**R***	AAAAAACCAAACCAAATTCTCC	

* Primters with 5´-biotin tag.

Supplementary Table S2. Comparison of clinicopathological features of all CRC cases

Clinical features		All cases	*BRAF*	*KRAS*	*NRAS*	No-mut	*P*-value (*K* vs *N* vs *No*)	*P*-value (*K* vs *N*)
# of sample		205	13	59	56	72		
Gender								
	Male	115	9	33	25	48	0.2	0.6
	Female	84	4	26	25	24		
	Unkown	6	0	0	6	0		
Age (y.o.)								
	Mean±SD	63.8±9.4	71.0±8.5	61.7±9.4	66.7±9.1	62.0±9.1	*0.005	*0.01
Tumor location								
	Proximal	61	13	23	6	17	*2×10^{-5}	*0.002
	Distal	137	0	36	43	55		
	Unkown	7	0	0	7	0		
Mucinous component								
	(+)	31	8	14	3	5	*0.004	*0.02
	(-)	167	5	45	46	67		
	Unkown	7	0	0	7	0		
AJCC Stage								
	I	15	3	0	11	0	*5×10^{-6}	*0.001
	II	54	5	17	16	16		
	III	64	3	21	11	26		
	IV	66	2	21	12	30		
	Unkown	6	0	0	6	0		
Lymph node metastasis								
	(+)	108	5	39	17	43	*0.005	*0.003
	(-)	87	8	20	30	28		
	Unkown	10	0	0	9	1		
Lymph vessel invasion								
	(+)	135	11	48	19	53	*5×10^{-6}	*8×10^{-6}
	(-)	63	2	11	30	19		
	Unkown	7	0	0	7	0		
Venous invasion								
	(+)	157	9	48	33	62	*0.04	0.1
	(-)	41	4	11	16	10		
	Unkown	7	0	0	7	0		
Microsatellite instability								
	MSI-H	17	11	3	0	3	0.3	0.3
	MSS	180	2	56	48	69		
	Unkown	8	0	0	8	0		
Methylation epigenotype								
	HME	13	9	2	0	2	*3×10^{-4}	*1×10^{-4}
	IME	89	4	40	18	27		
	LME	98	0	17	38	43		

No-mut: no mutation. *B vs K vs N*: *BRAF* vs *KRAS* vs *NRAS*. *K vs N*:*KRAS* vs *NRAS*. *MSI-H*: microsatellite instability high. *MSS*: microsatellite stable. *$P < 0.05$

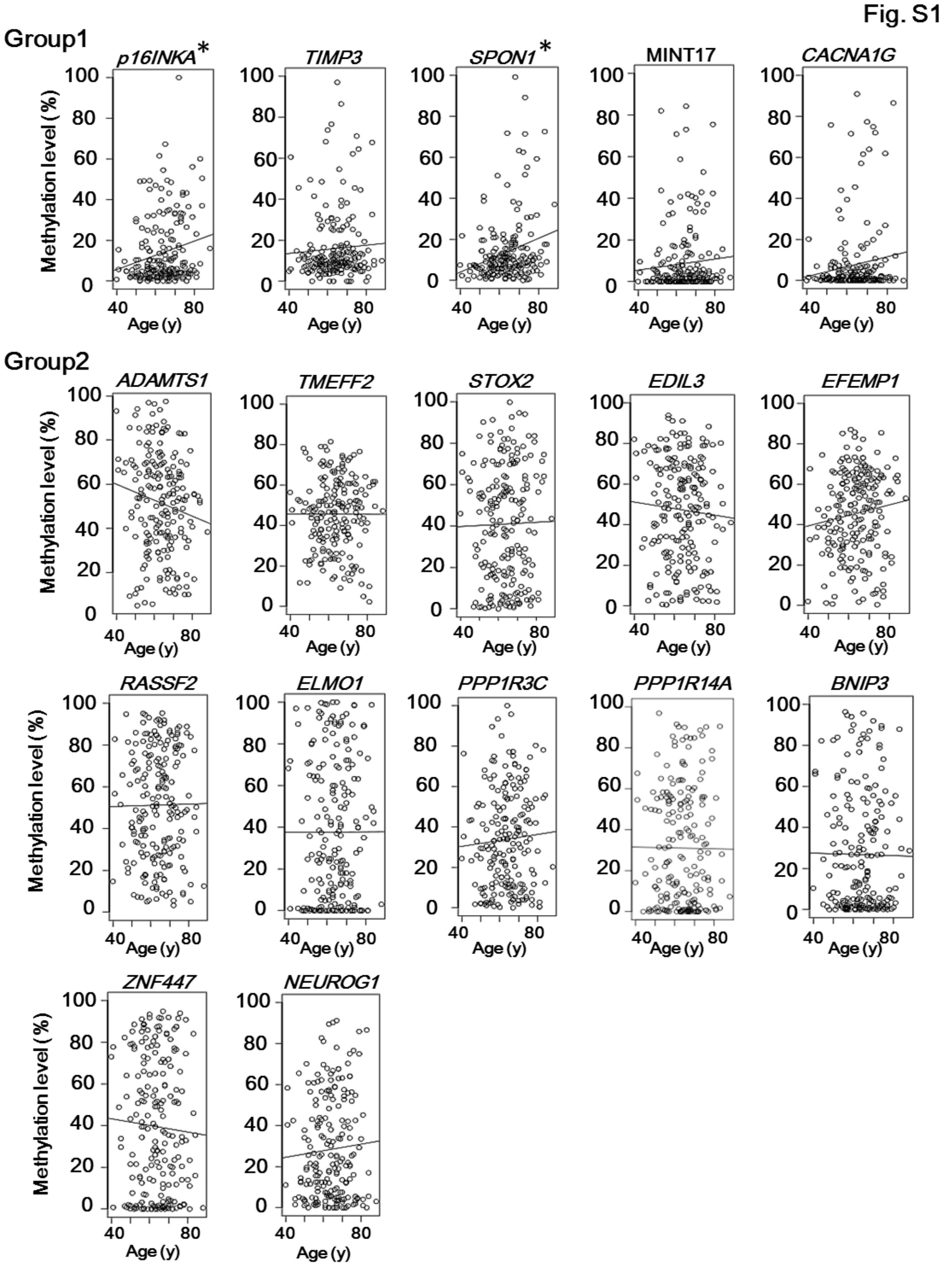

Supplementary Figure S1. Comparison between methylation levels and age using linear single regression model. Three of the six Group-1 markers showed a significant correlation between higher methylation levels and age (**P* < 0.008), while none of Group-2 markers did. Since six Group-1 markers were evaluated, *P*-values <0.008 (i.e., 0.05/6) were considered significant.

Fig. S2

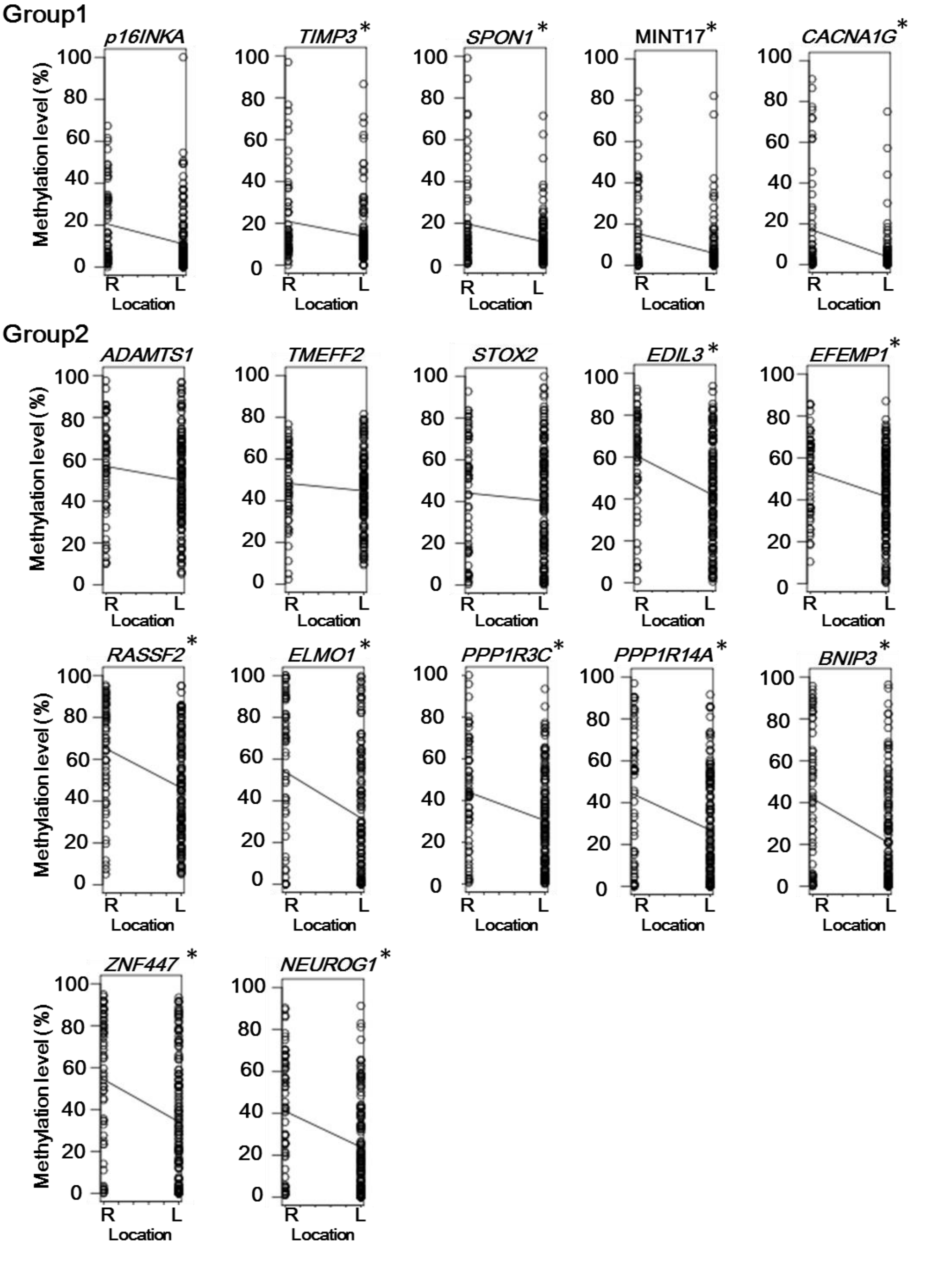

Supplementary Figure S2. Comparison between methylation levels and tumor location using a linear single regression model. While five of the Group-1 markers showed a significant correlation between higher methylation level and proximal location (*$P < 0.008$), 10 of 13 Group-2 markers showed significant correlation between higher methylation level and proximal location (*$P < 0.004$). Since six Group-1 markers and 13 Group-2 markers were evaluated, P-values <0.008 (i.e., 0.05/6) and <0.004 (i.e., 0.05/13), respectively, were considered significant.

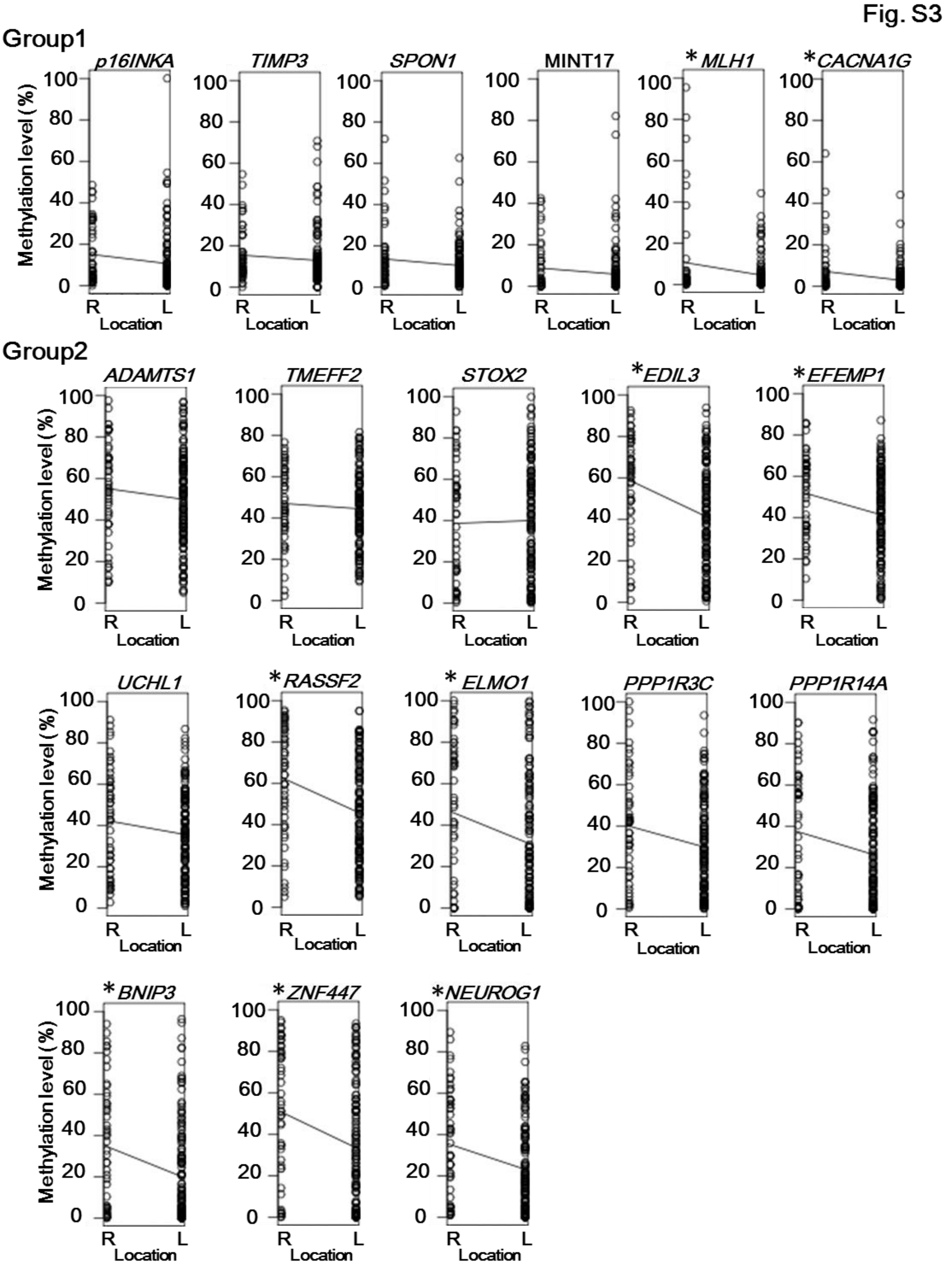

Supplementary Figure S3. Comparison between methylation levels and tumor location using a linear single regression model, excluding HME CRCs. While two of the Group-1 markers showed a significant correlation between higher methylation level and proximal location (*$P < 0.008$), 7 of 13 Group-2 markers showed a significant correlation between higher methylation level and proximal location (*$P < 0.004$). Since six Group-1 markers and 13 Group-2 markers were evaluated, *P*-values <0.008 (i.e., 0.05/6) and <0.004 (i.e., 0.05/13), respectively, were considered significant.
